# Questionnaire-based epidemiological survey of primary focal hyperhidrosis and survey on current medical management of primary axillary hyperhidrosis in Japan

**DOI:** 10.1007/s00403-022-02365-9

**Published:** 2022-06-29

**Authors:** Tomoko Fujimoto, Yuri Inose, Hideki Nakamura, Yoshinobu Kikukawa

**Affiliations:** 1 Ikebukuro Nishiguchi Fukurou Dermatology Clinic, 1-39-4 Nishi-Ikebukuro, Toshima-ku, Tokyo, Japan; 2 Maruho Co., Ltd., 1-5-22 Nakatsu, Kita-ku, Osaka, Japan

**Keywords:** Primary hyperhidrosis, Primary axillary hyperhidrosis, Japan, Epidemiological survey, Patient-reported outcome

## Abstract

To obtain current epidemiological information on primary focal hyperhidrosis in Japan, a large epidemiological survey was conducted using a web-based questionnaire. The prevalence of primary focal hyperhidrosis was 10.0% and the site-specific prevalence was highest for primary axillary hyperhidrosis (5.9%). The proportion of respondents with primary focal hyperhidrosis who had consulted a physician was 4.6%, which was similar to the low prevalence reported previously in 2013 in Japan. A questionnaire survey for physicians and individuals with primary axillary hyperhidrosis on the current medical management of primary axillary hyperhidrosis showed that physicians recognized the existence of patients who were very worried about hyperhidrosis, but failed to provide active treatment. Regarding the information provided by patients to physicians at presentation, it was found that patients failed to provide sufficient information to the physicians about their worries in daily life. Among individuals who had sought medical care with primary axillary hyperhidrosis, 62.3% reported that they were not currently receiving treatment, highlighting a challenge to be addressed regarding continued treatment. Frequently chosen options leading to willingness to receive treatment were less expensive treatment and highly effective treatment as well as feeling free to consult a physician, suggesting a desire for an improved medical environment.

## Introduction

Hyperhidrosis is a skin disease characterized by sweating in excess of that required for normal thermoregulation. Primary focal hyperhidrosis is idiopathic with bilateral excessive sweating localized to the palms, soles, axillae, or head/face [3, 5]. This condition negatively affects patients’ quality of life (QOL) by reducing work productivity and impairing mental health [2, 6, 8]. According to a report from a Japanese epidemiological survey in 2013, the prevalence of primary focal hyperhidrosis was 12.76%, and that of primary axillary hyperhidrosis was 5.75%, indicating that hyperhidrosis may reduce the QOL of many people. However, only 6.2% of respondents had visited medical institutions, suggesting that few patients who seek medical care receive appropriate treatment [4].

We present an epidemiological survey of primary focal hyperhidrosis in approximately 60,000 Japanese individuals, and a fact-finding survey in physicians and individuals with primary axillary hyperhidrosis to identify problems in the medical management of primary axillary hyperhidrosis.

## Materials and methods

### Ethical conduct and informed consent

This study was conducted with the approval of the Ethical Review Board of Kojinkai, Association of Medical Corporation Hosui General Medical Clinic (Approved November 19, 2020). The study presented Internet-based questionnaire to individuals and physicians registered panels of the questionnaire survey companies INTAGE Inc. (Tokyo, Japan) and INTAGE Healthcare Inc. (Tokyo, Japan), respectively. The study was initiated after informed consent was obtained from subjects who confirmed the outline of the study and publishing their data on the website. Individuals were aged 15 years and older were registered in the panel, and minors were enrolled in the study with their personal consent and the approval of their legal guardians.

### Epidemiological survey of primary focal hyperhidrosis

A survey, conducted from December 7 to December 11, 2020, calculated the prevalence of primary focal hyperhidrosis, the proportion who had consulted a physician, and the proportion who were consulting a physician. With a target sample size of approximately 59,900 respondents reflecting the composition of the Japanese population in terms of sex and age, those who satisfied all the following criteria based on the self-report were considered to have primary focal hyperhidrosis: (1) currently having excessive sweating in the axillae, head/face, palms, or soles; (2) meeting at least two of the diagnostic criteria for primary focal hyperhidrosis (Table 1) [5, 7]; and (3) having no symptoms of secondary hyperhidrosis.

**Table 1:** Diagnostic criteria for primary focal hyperhidrosis

1) Bilateral and relatively symmetrical sweating
2) Symptoms of excessive sweating impairs daily activities
3) Frequency of at least one episode of excessive sweating per week
4) Age of onset less than 25 years
5) Positive family history
6) Cessation of focal sweating during sleep

#### Epidemiological survey items

1. Age
2. Sex
3. Presence or absence of previous excessive sweating in the axillae, head/face, palms, and/or soles, and presence or absence of current excessive sweating in any of these sites
4. Meeting any of the diagnostic criteria for primary focal hyperhidrosis (Table 1) [5, 7] or not
5. Having consulted a physician with a diagnosis of hyperhidrosis or not; and consulting a physician with a diagnosis of hyperhidrosis or not
6. Having any symptom of secondary hyperhidrosis or not; excessive sweating due to disease under treatment; heavy sweating during sleep; unilateral excessive sweating; excessive sweating due to prescription drug(s); and excessive sweating due to menopausal symptoms

Multiple answers were allowed for survey items 3, 4, and 6.

### Survey on current medical management of primary axillary hyperhidrosis

#### Survey of physicians

From December 8 to December 14, 2020, a survey of physicians who satisfied all the following criteria was performed: (1) primary medical specialty of dermatology, plastic/cosmetic surgery, general internal medicine, neurology, psychosomatic medicine, or psychiatry; and (2) at least one patient with primary focal hyperhidrosis was seen in the last year.

The total target sample size was 450 respondents: 300 from dermatology, 50 from plastic/cosmetic surgery, and 100 from other specialties.

#### Survey items for physicians

1. Demographics of physicians (primary medical specialty, management style, and number of patients with primary focal hyperhidrosis seen in the last year)
2. Impression of hyperhidrosis
3. Primary axillary hyperhidrosis to be treated
4. Criteria used to assess the severity of primary axillary hyperhidrosis
5. Number of patients by hyperhidrosis disease severity scale (HDSS) [15] score
6. Each number of patients with HDSS 2 to 4 experienced episode(s) due to primary axillary hyperhidrosis in the opinion of the physician
7. Information provided by patients at presentation

Multiple answers were allowed for survey items 2, 4, and 7.

Survey item 4 queried, the patient-reported outcome (PRO) in the criteria used to assess the severity of primary axillary hyperhidrosis. In this study, a version of the Axillary Hyperhidrosis Patient Measures (AHPM), a primary axillary hyperhidrosis-specific PRO [12, 13], partially modified for Japanese was used. In survey item 6, all questions about each episode experienced related to primary axillary hyperhidrosis were asked using the six Weekly Impact (WI) items in the AHPM [12].

#### Survey of individuals with primary axillary hyperhidrosis

A survey was conducted from December 18 to December 24, 2020. With a target sample size of 500 respondents, people who satisfied all the following criteria based on the self-report were included as individuals with primary axillary hyperhidrosis, including those who had sought medical care: (1) reporting (previous or current) excessive sweating in the axillae; (2) meeting at least two of the diagnostic criteria for primary focal hyperhidrosis (Table 1) [5, 7]; (3) having no symptom of secondary hyperhidrosis; and (4) having no symptom of underarm odor (wet ear wax or family member with underarm odor).

Based on the pre-screening results, the survey included all respondents who had sought medical care and with an HDSS of 4 for axillary hyperhidrosis among those had not sought medical care, and 150 respondents each with an HDSS of 1 to 3 for axillary hyperhidrosis among those who had not sought medical care.

#### Survey items for individuals with primary axillary hyperhidrosis

1. Age, sex, having sought medical care with a diagnosis of hyperhidrosis or not
2. HDSS score when sweat began to trouble the respondent
3. Episodes experienced when sweat began to trouble the respondent
4. Information that the respondent who had sought medical care provided to a physician at presentation
5. Drugs/treatments that have been given to the respondent who sought medical care, drugs/treatments that are currently given to the respondent who sought medical care
6. Reasons why the respondent has never sought medical care
7. What the respondent who sought medical care expects of future treatment
8. What will motivate a respondent who has never sought medical care to visit a health care facility

Multiple answers were allowed for survey items 3 to 8.

In survey item 3, all questions about episodes experienced when sweat began to trouble the respondent were asked using the six WI items in the AHPM [12].

## Results

### Epidemiological survey of primary focal hyperhidrosis

An epidemiological survey of primary focal hyperhidrosis was conducted in 60,969 individuals. The target sample size was not achieved for males and females aged 15 to 19 years, males aged 20 to 29 years, or males aged 30 to 39 years; however, the target sample size was exceeded for females aged 20 to 29 years and 30 to 39 years (Table 2).

**Table 2:** No. of subjects (epidemiological survey)

Age (years)		15–19	20–29	30–39	40–49	50–59	60–69
Total	60,969	2184	9456	11,696	13,552	12,348	11,733
Male	26,426	493	2185	4878	6868	6226	5776
Female	34,543	1691	7271	6818	6684	6122	5957

To investigate the epidemiology of primary focal hyperhidrosis, the prevalence and proportion of those who had consulted a physician, and the proportion of those who were consulting a physician were calculated (Table 3). The prevalence of primary focal hyperhidrosis was 10.0%. By site, the prevalence was highest in the axillae (5.9%), followed by the head/face (3.6%), palms (2.9%), and soles (2.3%). The proportion of individuals with primary focal hyperhidrosis who had consulted a physician was 4.6%. By site, the proportion was highest in the soles (10.2%), followed by the palms (9.6%), axillae (4.4%), and head/face (3.5%). The proportion of individuals with primary focal hyperhidrosis who were consulting a physician was 0.7%.

**Table 3:** Prevalence of primary focal hyperhidrosis, and proportion of individuals who have consulted or are consulting a physician

		No. of individuals with current excessive sweating	Prevalence^a^ (%)	No. of individuals who have consulted a physician for hyperhidrosis	Proportion (%) who have consulted a physician^b^	No. of individuals who are consulting a physician for hyperhidrosis	Proportion (%) who are consulting a physician^c^
Primary focal hyperhidrosis		6116	10.0	278	4.6	45	0.7
Site	Axilla	3576	5.9	157	4.4	25	0.7
	Head/face	2194	3.6	76	3.5	17	0.8
	Palm	1769	2.9	169	9.6	21	1.2
	Sole	1369	2.3	140	10.2	28	2.1

^a^Proportion of individuals with current excessive sweating among all respondents (*n* = 60,969)

^b^Proportion of individuals who have consulted a physician with a diagnosis of hyperhidrosis among all respondents with current excessive sweating

^c^Proportion of individuals who are consulting a physician with a diagnosis of hyperhidrosis among all respondents with current excessive sweating

The prevalence of primary focal hyperhidrosis (including site-specific prevalence) was calculated by sex and age (Table 4). The prevalence of primary focal hyperhidrosis was 9.0% and 10.9% in males and females, respectively. By site, the sex ratio was highest in the axillae, with the prevalence being 1.7 times higher in females than in males. A higher prevalence in males than in females was observed only for the head/face. By age, the prevalence of primary focal hyperhidrosis was ≥ 10% at 20 to 49 years, with a peak at 20 to 39 years.

**Table 4:** Prevalence of primary focal hyperhidrosis (by sex and age)

No. of individuals with current excessive sweating and prevalence (%)										
		Sex		Ratio^a^	Age					
		Male	Female		15–19 years	20–29 years	30–39 years	40–49 years	50–59 years	60–69 years
Primary focal hyperhidrosis		2369 (9.0)	3747 (10.9)	1.2	217 (9.9)	1285 (13.6)	1572 (13.4)	1463 (10.8)	909 (7.4)	670 (5.7)
Site	Axilla	1115 (4.2)	2461 (7.1)	1.7	104 (4.8)	807 (8.5)	1035 (8.9)	882 (6.5)	434 (3.5)	314 (2.7)
	Head/face	1111 (4.2)	1083 (3.1)	0.7	58 (2.7)	333 (3.5)	481 (4.1)	561 (4.1)	422 (3.4)	339 (2.9)
	Palm	712 (2.7)	1057 (3.1)	1.1	124 (5.7)	535 (5.7)	490 (4.2)	357 (2.6)	186 (1.5)	77 (0.7)
	Sole	570 (2.2)	799 (2.3)	1.1	63 (2.9)	392 (4.2)	395 (3.4)	289 (2.1)	163 (1.3)	67 (0.6)

^a^Female/male ratio of prevalence

### Survey on the current medical management of primary axillary hyperhidrosis

Questionnaire surveys of the current medical management of primary axillary hyperhidrosis were conducted in physicians as well as individuals who satisfied the diagnostic criteria for primary axillary hyperhidrosis (individuals with primary axillary hyperhidrosis). Subject demographics are presented in Table 5.

**Table 5:** Subject demographics (survey of current medical management of primary axillary hyperhidrosis)

Attributes of physicians (*n* = 493)		
Primary medical specialty^a^ *n* (%)	Dermatology	333 (67.5)
	Plastic/cosmetic surgery	85 (17.2)
	Other	117 (23.7)
Management style *n* (%)	Hospital	309 (62.7)
	Clinic	184 (37.3)
Mean number of patients with primary focal hyperhidrosis seen in the last year		10.1

Attributes of individuals with primary axillary hyperhidrosis (*n* = 676)		
		*n* (%)
Age	15–19 years	26 (3.8)
	20–29 years	147(21.7)
	30–39 years	173 (25.6)
	40–49 years	167 (24.7)
	50–59 years	92 (13.6)
Sex	Male	232 (34.3)
	Female	444 (65.7)
Have sought medical care	Yes	77 (11.4)
	No	599 (88.6)

^a^Cosmetic dermatology includes dermatology and plastic/cosmetic surgery

In response to a question about the impression of hyperhidrosis, physicians responded as follows: some patients are very worried (69.8%); active treatment cannot be provided because of limited treatment options (58.2%); the disease should be treated by a dermatologist (34.1%); the disease is a low priority because of the small number of patients (18.9%); and the disease can be managed by self-care for most patients (3.9%) (data not shown).

In response to a question about primary axillary hyperhidrosis to be treated, physicians responded as follows: HDSS 2 or higher (27.8%); HDSS 3 or higher (32.3%); treated if a patient is worried (38.1%); and hyperhidrosis itself should not be treated (1.8%) (data not shown).

In response to a question querying the criteria, physicians used to assess the severity of primary axillary hyperhidrosis, the most common response was “no assessment criteria used” (50.1%), followed by HDSS (43.4%), PRO measures in primary axillary hyperhidrosis (14.8%), and other criteria (0.2%) (data not shown).

**Fig. 1:**
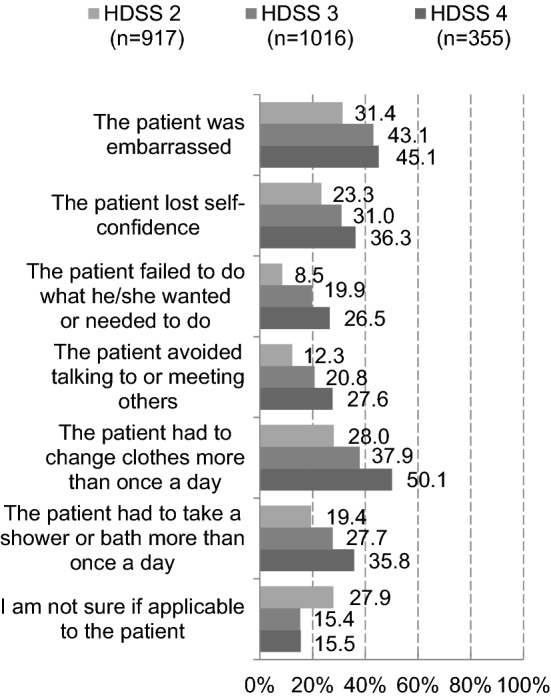
Episodes of patients with primary axillary hyperhidrosis assessed by physicians (reported by physicians)

Physicians were asked about the total and HDSS score-specific numbers of patients with primary axillary hyperhidrosis seen in the last year to calculate the proportion of patients with each HDSS score, as follows: 9.0% for HDSS 1, 36.5% for HDSS 2, 40.4% for HDSS 3, and 14.1% for HDSS 4 (data not shown). In addition, the proportion of patients with HDSS 2 to 4 who experienced individual episodes increased as the HDSS score increased (Fig. 1).

**Fig. 2:**
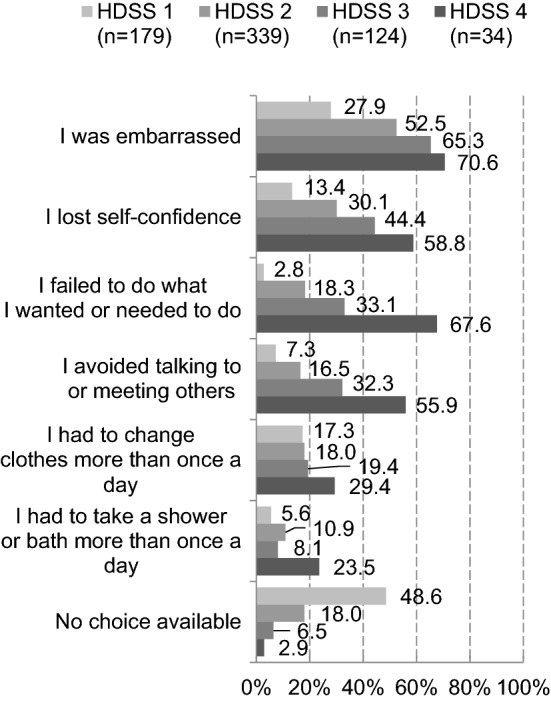
Episodes experienced when sweat began to trouble the respondent (reported by individuals with primary axillary hyperhidrosis)

In response to a question about the HDSS score when sweat began to trouble the respondent, individuals with primary axillary hyperhidrosis responded as follows: HDSS 1 in 26.5%, HDSS 2 in 50.1%, HDSS 3 in 18.3%, and HDSS 4 in 5.0% (data not shown). In addition, episodes experienced when sweat began to trouble the respondent were investigated by HDSS score. The following episodes were more often experienced by respondents with greater HDSS severity: being embarrassed, loss of confidence, failure to do what the respondent wanted or needed to do, and avoidance of talking to or meeting others. “No choice available” was chosen by approximately half (48.6%) of respondents with HDSS 1 and 18% or fewer of respondents with HDSS 2 or higher (Fig. 2).

**Fig. 3:**
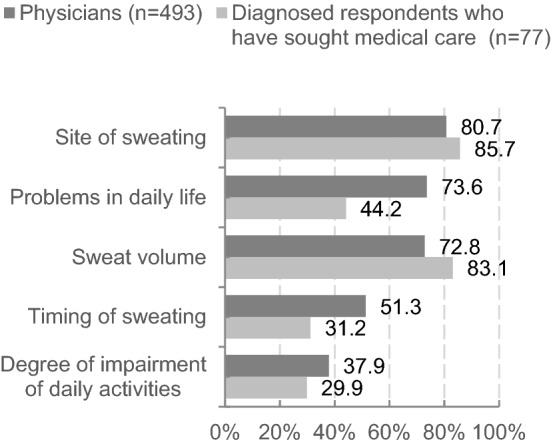
Information provided by patients/provided to physicians at presentation (reported by physicians/diagnosed respondents who have sought medical care)

Physicians and diagnosed respondents who had sought medical care were asked about information provided by patients and provided to physicians at presentation, respectively. The most common information that physicians received was the site of sweating (80.7%), followed by problems in daily life (73.6%), and sweat volume (72.8%). The most common information that diagnosed respondents who had sought medical care provided was the site of sweating (85.7%), followed by sweat volume (83.1%), and problems in daily life (44.2%). This demonstrated a difference in providing information about “problems in daily life” (Fig. 3).

**Fig. 4:**
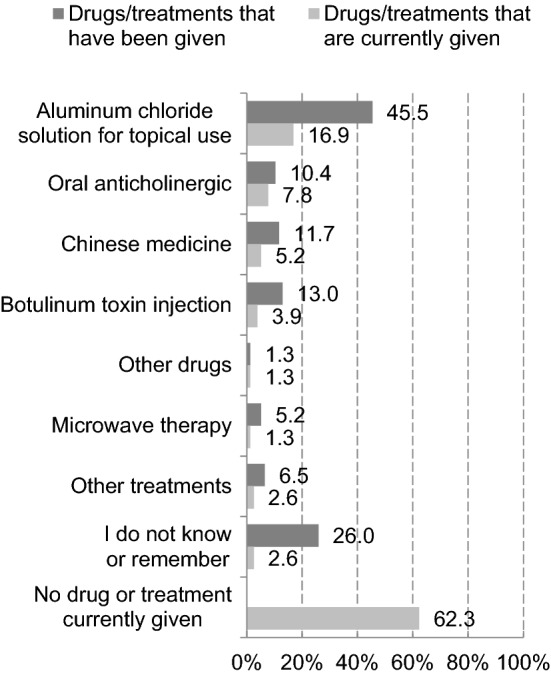
Drugs/treatments that have been given and that are currently given (reported by diagnosed respondents who have sought medical care, *n* = 77)

Diagnosed respondents who had sought medical care were asked about the drugs/treatments that had been given and drugs/treatments that were currently given. For diagnosed respondents, the most common drug/treatment that had been given was aluminum chloride solution for topical use (45.5%). However, 62.3% were currently given no drug or treatment (Fig. 4).

Responses to a question about why the respondent had never sought medical care were as follows: treatment may be expensive (35.9%); I do not consider sweating a disease or illness (34.2%); sweating can be controlled by commercially available antiperspirant products (26.4%); I do not know which hospital to visit (22.5%); sweating does not require consultation (18.9%); I am embarrassed to seek medical care (17.9%); I give up (17.4%); I did not know that sweating should or could be treated (14.0%); I am too busy (10.0%); and the treatment is uncomfortable (6.8%) (data not shown).

**Fig. 5:**
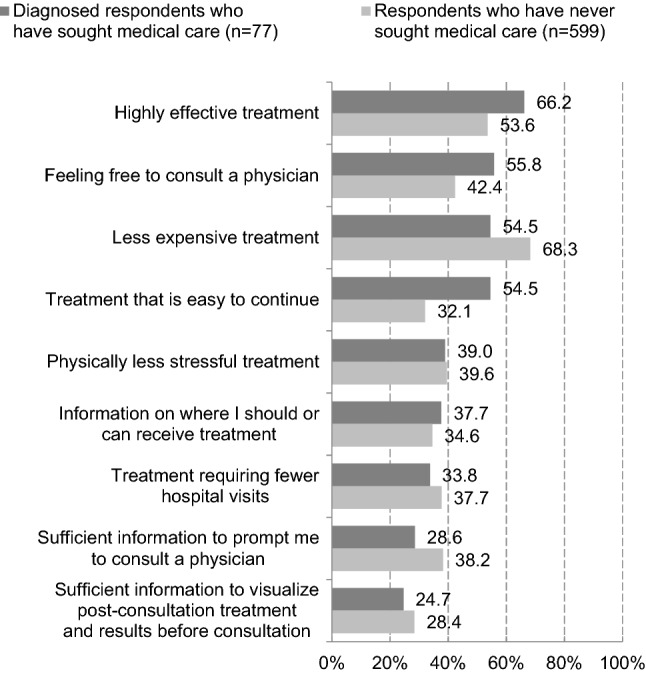
What diagnosed respondents who have sought medical care expect for future treatment and what will motivate respondents who have never sought medical care to visit a health care facility

The most common response to a question about what the diagnosed respondent who had sought medical care expected from future treatment was highly effective treatment (66.2%), followed by feeling free to consult a physician (55.8%), less expensive treatment (54.5%), and treatment that is easy to continue (54.5%). The most common response to a question about what would motivate a respondent who had never sought medical care to visit a health care facility was less expensive treatment (68.3%), followed by highly effective treatment (53.6%), and feeling free to consult a physician (42.4%) (Fig. 5).

## Discussion

A large epidemiological survey was conducted using a web-based questionnaire to obtain current epidemiological information on primary focal hyperhidrosis in Japan. Studies from various countries have reported the prevalences of (primary) hyperhidrosis ranging from 4.8 to 14.5% [3, 11, 14]. According to previous surveys, the prevalence of primary focal hyperhidrosis in Japan was 12.76% [4], by site, primary axillary hyperhidrosis was most prevalent (5.75%), similar to the current survey findings. Although the mean age of onset of primary palmar hyperhidrosis was reported to be less than 15 years previously, individuals aged 15 years and older were included in the present survey, possibly resulting in the lower prevalence of primary palmar hyperhidrosis.

Although 27 to 51% of hyperhidrosis sufferers in Germany and the USA had consulted a physician [1, 3], the proportion of sufferers in the present survey who had consulted a physician was low at 4.6%, similar to that previously reported (6.2%) [4]. This was lower than the proportion of patients who sought medical care for acne in Japan, which was also low (16.2%) [17]. By site, the proportion who had consulted a physician for the palms and soles was at least twice as high as those who had consulted a physician for the axillae and head/face, indicating that those with excessive sweating in the palms and soles were more worried. In addition, the proportion who were consulting a physician was 0.7%, indicating that the majority failed to continue treatment.

Previous studies reporting the sex ratio of the prevalence of primary focal hyperhidrosis [1, 11, 16], and a previous report in Japan [4] showed a significantly higher prevalence in males. In the present survey, the overall prevalence did not differ greatly between males and females, but the site-specific prevalence of primary axillary hyperhidrosis was 1.7 times higher in females. Only primary focal hyperhidrosis of the head/face had a higher prevalence in males than females. A previous study reported that males had the highest prevalence of primary focal hyperhidrosis of the head [4] suggesting sex differences might be site specific, although further investigation is needed.

By age, the prevalence of primary focal hyperhidrosis peaked at 20 to 39 years. According to previous reports [3, 4], sweating affects daily life, especially in this generation.

We conducted a survey among physicians and individuals with primary focal hyperhidrosis to identify problems in the medical management of primary axillary hyperhidrosis. This study found that physicians recognized patients who were very worried about hyperhidrosis, but failed to provide active treatment because of limited treatment options, indicating novel treatment options are required.

The most common response to a question about the criteria used to assess the severity of primary axillary hyperhidrosis was “no assessment criteria used.” The HDSS, the most commonly used assessment criteria, is easy to use for assessment, but is not suitable for understanding the depth of patient worry because it uses four broad categories. Hyperhidrosis-specific (or primary axillary hyperhidrosis-specific) PRO measures [9, 10, 12] have been reported and may provide a more specific assessment of symptoms. In the present survey, the PRO [12] was rarely used by physicians, indicating that recognition of their use as an assessment method was low.

Respondents were asked about episodes experienced when sweat began to trouble them, and the results were reviewed by HDSS score. Psychiatric episodes were more often experienced as the HDSS score increased. In the physician survey, a similar relationship was observed between HDSS score and episodes experienced. The PRO measures, such as the six WI items used to query experiences of sweating-related episodes, may be useful in understanding the depth of patient worry and complement the HDSS assessment. In addition, physicians and diagnosed respondents were asked about information provided by patients to physicians at presentation. The results indicated that patients failed to provide sufficient information to physicians about their condition, especially problems in daily life. The PRO measures, such as the six WI items, are expected to improve understanding of patient worry and enhance communication between patients and physicians.

In the physician survey, there were conflicting responses to a question about primary axillary hyperhidrosis to be treated: HDSS 3 or higher (32.3%) vs HDSS 2 or higher (27.8%) vs patient complaint (38.1%). In Fig. 2, the largest difference between respondents with HDSS 1 and those with HDSS 2 was observed for those who chose “no choice available.” More than 80% of respondents with HDSS 2 experienced many of these episodes, indicating that even these respondents were worried about sweating. Although hyperhidrosis to be treated differed among physicians, respondents with HDSS 2 or higher sought medical care in practice (the proportion of patients with HDSS 2 or HDSS 3 seen by physicians in the last year accounted for approximately 80% of all patients), and more than 80% of those with HDSS 2 or higher were worried about sweating, suggesting that those with HDSS 2 should be treated. Because it is difficult to make a clear distinction between HDSS 2 and HDSS 3, it is important for physicians to understand how seriously patients are worried using the PRO measures for primary axillary hyperhidrosis and to provide suitable therapeutic interventions.

In diagnosed respondents, the most common drugs/treatments given was aluminum chloride solution for topical use, indicating this might be the main treatment. However, more than 60% of respondents failed to continue treatment, suggesting a challenge to be addressed regarding the current treatment. In particular, the continued use of topical aluminum chloride solution may be difficult for some patients because of skin irritation.

In addition, respondents who had never sought medical care may have misunderstood that excessive sweating is not a medical condition to be treated or that it is expensive to treat, although they hoped for highly effective treatment. In respondents who had sought medical care, it is desirable to improve the medical care environment in health care facilities.

In summary of the questionnaire survey of the current medical management of primary axillary hyperhidrosis, patients failed to provide sufficient information about themselves to physicians, especially regarding problems in daily life. We consider that the adoption of PRO would be beneficial as a tool to allow physicians to enhance their communication with patients and thereby better understand their worries. In addition, we found that neither physicians nor individuals with primary axillary hyperhidrosis were satisfied with the current countermeasures or treatments for primary axillary hyperhidrosis. This highlights the need to increase treatment options and improve the environment for physicians and patients, for example, by making it easier for those worried about excessive sweating to consult physicians and helping people understand that excessive sweating is a disease that should be treated in a health care facility.

## Data Availability

Upon reasonable request.
